# Supplementary material for: Resolvin-D2 targets myogenic cells and improves muscle regeneration in Duchenne muscular dystrophy
Source: Nat Commun. 2021 Oct 29;12:6264. doi: 10.1038/s41467-021-26516-0 (PMC8556273; doi:10.1038/s41467-021-26516-0)
Supplement: Supplementary file 1 — Supplementary Information [file 41467_2021_26516_MOESM1_ESM.pdf]

Supplementary material for

Resolvin-D2 targets myogenic cells and improves muscle regeneration in Duchenne Muscular Dystrophy

Junio Dort, Zakaria Orfi, Paul Fabre, Thomas Molina, Talita C. Conte, Karine Greffard, Ornella Pellerito, Jean-François Bilodeau, and Nicolas A. Dumont

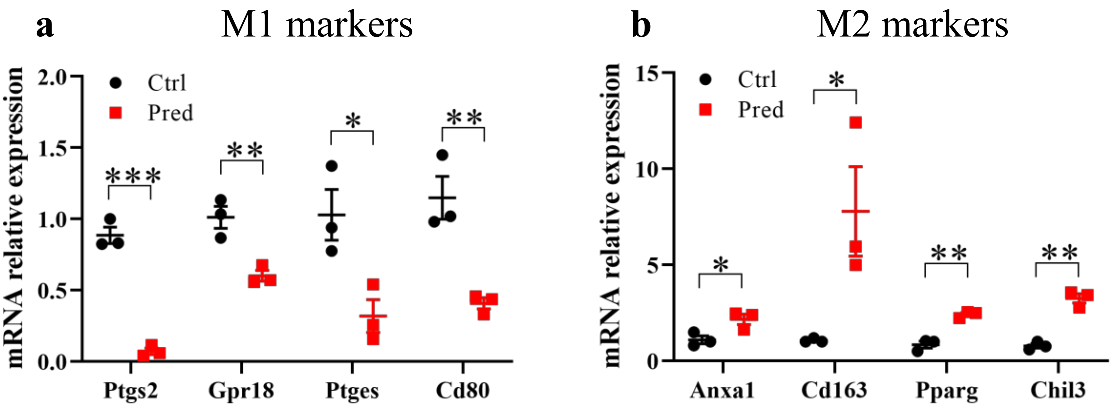

Supplemental Figure 1. Impact of prednisone on the phenotype of M1-polarized macrophages. Monocytes purified from bone marrow of *mdx* mice are differentiated into macrophages (M-CSF), polarized into M1 macrophages (IFN-γ and LPS supplementation) and treated with prednisone (pred; 10 μM) or vehicle (Ctrl) for 48 h. Gene expression of the (**a**) pro-inflammatory markers *Ptgs2* ($p = 0.000193$), *Gpr18* ($p = 0.008992$), *Ptges* ($p = 0.028523$), and *Cd80* ($p = 0.008745$), and (**b**) anti-inflammatory markers *Anxa1*($p = 0.034402$), *Cd163* ($p = 0.044976$), *Pparg* ($p = 0.001449$), and *Chil3* ($p = 0.000758$) on M1-polarized macrophages treated with or without prednisone for 48 h. Data are presented as mean ± SEM, n = 3 biologically independent samples performed in technical duplicates and analyzed with two-tailed unpaired Student's t-test. All data were analyzed with a 95% confidence interval. *$p < 0.05$, **$p < 0.01$, ***$p < 0.001$.

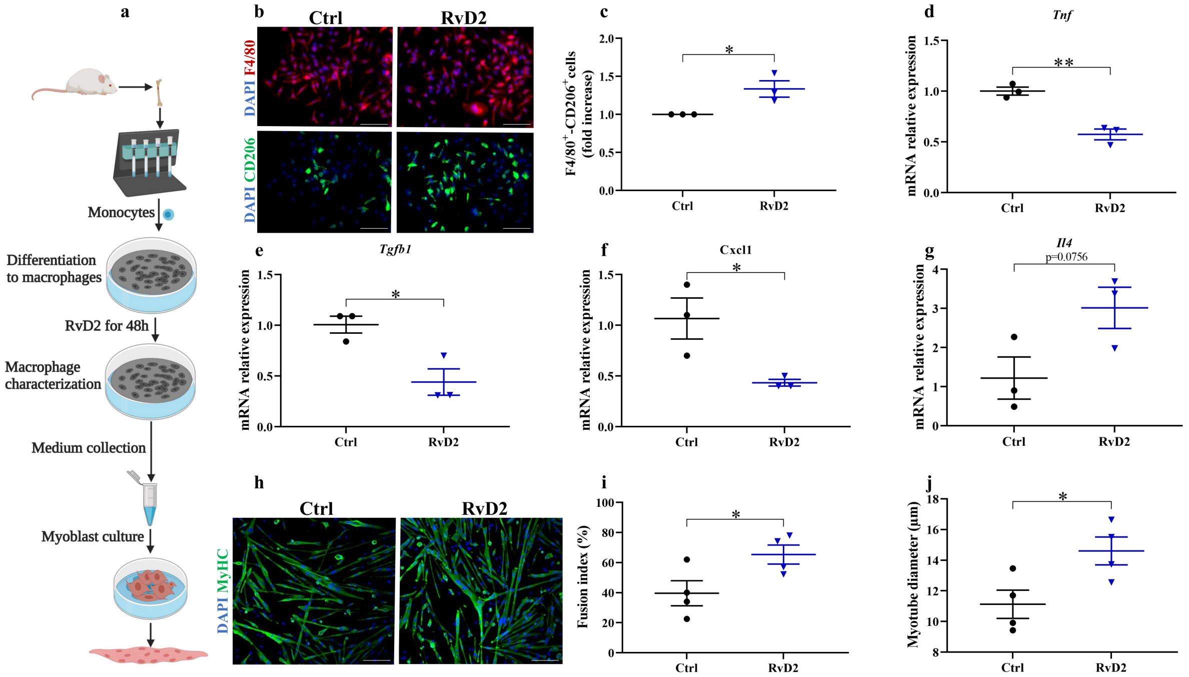

Supplemental Figure 2. Resolvin-D2 promotes the anti-inflammatory phenotype of unpolarized macrophages and their release of pro-myogenic factors. **a** Graphical overview of the myoblast:macrophage-conditioned medium co-culture experiments (created with BioRender.com). **b** Representative images of immunofluorescence performed on cultured macrophages for F4/80 (pan-macrophage marker; red), CD206 (anti-inflammatory macrophage marker; green), and DAPI (blue) Scale bars = 50 µm. **c** Percentage of anti-inflammatory macrophages ($F4/80^{+}CD206^{+}$ / total $F4/80^{+}$) following 48 h treatment with or without Resolvin-D2 (RvD2, 200 nM) (p = 0.0360). **d-g** qPCR analyses performed on macrophages treated with or without RvD2 for 48 h, to determine their expression of (**d**) Tumour Necrosis Factor alpha (*Tnf*) (p = 0.0030), (**e**) Transforming Growth Factor beta (*Tgfb1*) (p = 0.0214), (**f**) Chemokine (C-X-C motif) ligand 1 (*Cxcl1;* IL-8 homolog) (p = 0.0369), and (**g**) Interleukin 4 (*Il4*) (p = 0.0756). **h** Representative images of primary myoblasts differentiated into myotubes for 4 days with the macrophage-conditioned medium and stained for myosin heavy chain (MyHC, green) and DAPI (blue) Scale bars = 75 µm. **i** Quantification of the fusion index (proportion of nuclei into multinucleated myotubes / total nuclei) (p=0.0486) and (**j**) myotube diameter (p = 0.0359). Data are presented as mean ± SEM, n = 3 (panels **c-g**) or n = 4 (panels **i**,**j**) biologically independent samples performed in technical duplicates and analyzed with the two-tailed unpaired Student's t-test. All data were analyzed with a 95% confidence interval. *$p < 0.05$, **$p < 0.01$.

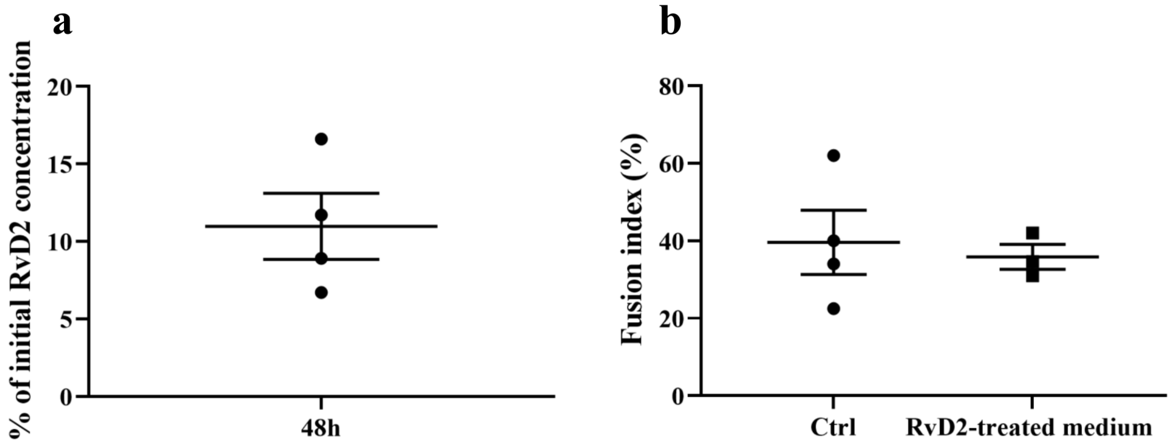

Supplemental Figure 3. Myogenic effects of macrophage-conditioned medium are not mediated by remaining Resolvin-D2 in the medium. a Concentration of Resolvin-D2 (RvD2) was assessed by ELISA in the culture medium 48 h after the supplementation of RvD2. The final concentration was expressed as the percentage of the initial concentration (200 nM). **b** To determine if the low remaining level of RvD2 in the medium after 48 h of treatment affects myogenesis, we added RvD2 in the medium in a macrophage-free well for 48 h. The medium was added to differentiating myoblasts. Data showed no difference compared to control, indicating that the myogenic effect of the macrophage-conditioned media is driven by paracrine factors secreted by macrophages upon RvD2 treatment and not by remaining RvD2 in the medium. Data are presented as mean ± SEM, n = 4 (except n = 3 for RvD2-treated medium group in panel **b**) biologically independent samples performed in technical duplicates. Data were analyzed with two-tailed unpaired Student's t-test with a 95% confidence interval.

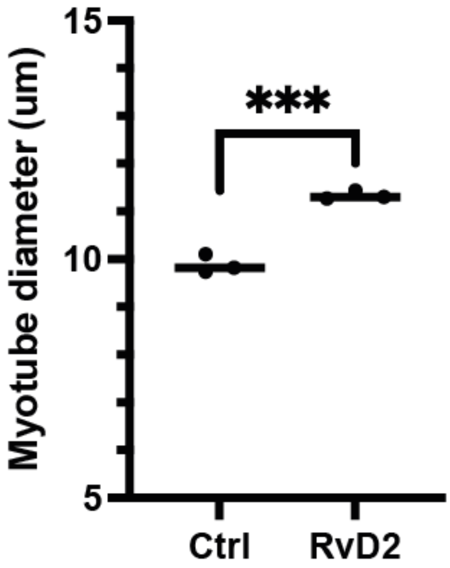

Supplemental Figure 4. Effect of RvD2 on myotubes. Myotubes were differentiated for 2 days. Thereafter, Resolvin-D2 (RvD2, 200 nM) or vehicle (Ctrl) was added in the differentiating medium for an additional 2 days, and myotube diameter was measured (p = 0.000278). Data are presented as mean ± SEM; n=3 biologically independent samples performed in technical duplicates and analyzed using two-tailed unpaired Student's t-test. All data were analyzed with a 95% confidence interval. ***p < 0.001, compared to vehicle.

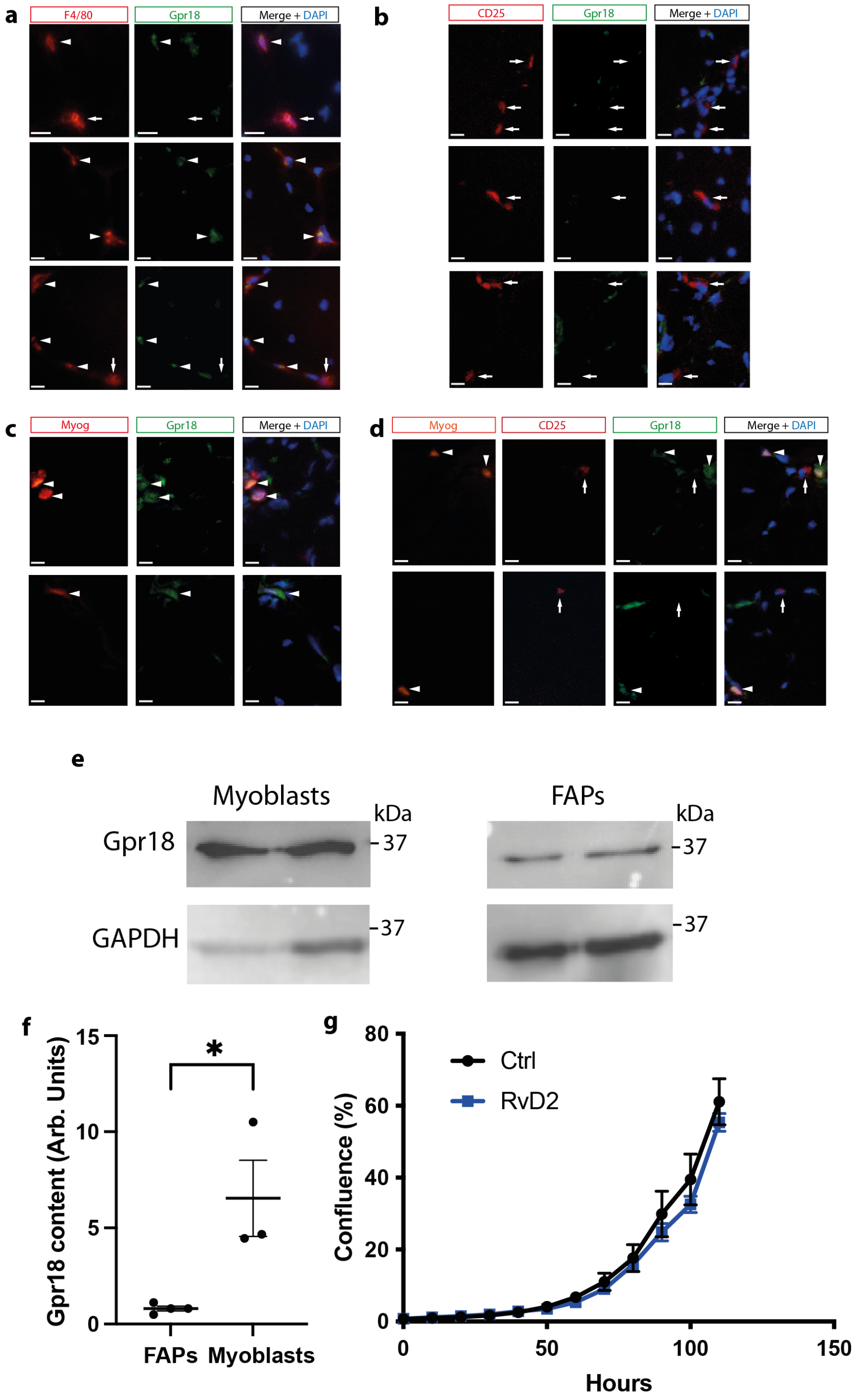

Supplemental Figure 5. Gpr18 expression in different cell types in skeletal muscle. Co-immunofluorescence of Gpr18 (RvD2 receptor) and markers for different cell types was performed on *tibialis anterior* (TA) muscles of *mdx* mice. **a** Representative pictures of F4/80 (macrophage marker; red), Gpr18 (green), and DAPI (blue). Scale bars = 10 µm. Arrowheads show double positive cells ($F4/80^+$ $Gpr18^+$; M1 macrophages) and arrows identify $F4/80^+$ $Gpr18^-$ cells (M2 macrophages). **b** Representative pictures of CD25 (Treg marker; red), Gpr18 (green), and DAPI (blue). Scale bars = 10 µm. Virtually all CD25 cells are negative for Gpr18

as identified by white arrows. **c** Representative pictures of Myogenin (Myog, differentiated myoblast marker; red), Gpr18 (green), and DAPI (blue). Scale bars = 10 μm. Arrowheads show double positive cells ($Myog^{+}$ $Gpr18^{+}$). **d** Representative pictures of Myog (orange), CD25 (red), Gpr18 (green), and DAPI, showing that $Myog^{+}$ cells express Gpr18 (arrowheads) but not $CD25^{+}$ cells (arrows). Scale bars = 10 μm. **e**,**f** Representative Western blots and quantification of the expression of Gpr18 (Gpr18/GAPDH) on proliferative myoblasts and FAPs isolated from *mdx* mice and cultured *in vitro* (p=0.0183). The samples derive from the same experiment and the gels/blots were processed in parallel. **g** Growth curves of FAPs treated with RvD2 (200 nM) or vehicle. **f**,**g** Data are presented as mean ± SEM; n = 3 (except for FAPs in panel **f**, n = 4) biologically independent samples performed in technical duplicates and analyzed with the two-tailed unpaired Student's t-test. All data were analyzed with a 95% confidence interval. *$p < 0.05$.

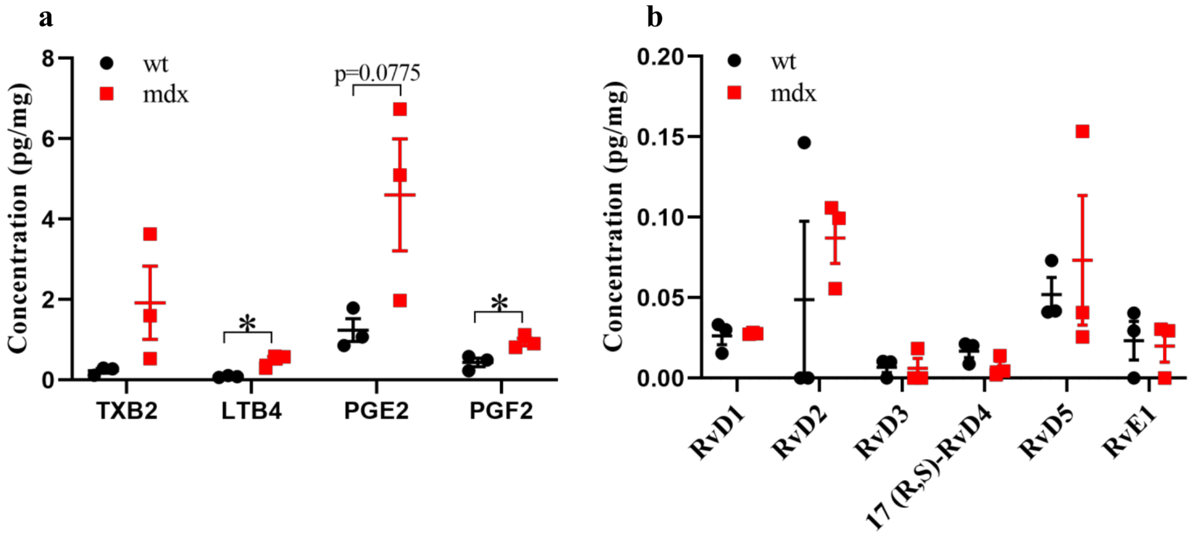

Supplemental Figure 6. Expression of bioactive lipids in dystrophic muscles. Mass spectrometry (LC-MS/MS) analysis of pro-inflammatory and pro-resolving bioactive lipids levels (pg/mg of muscle) in the gastrocnemius muscle of *mdx* and wild-type (wt) mice. **a** Pro-inflammatory mediators: TXB_2 (thromboxane-B_2), LTB_4 (Leukotriene-B_4, $p = 0.014304$), PGE_2 (Prostaglandin-E_2, $p = 0.077514$), $PGF_{2\alpha}$ (Prostaglandin-$F_{2\alpha}$, $p = 0.022919$). **b** Pro-resolving mediators: Resolvin-D1 (RvD1), RvD2, RvD3, 17(*R,S*)-RvD4, RvD5, Resolvin-E1 (RvE1). Data are presented as mean ± SEM; n = 3 biologically independent samples performed in technical duplicates and analyzed with the two-tailed unpaired Student's t-test. All data were analyzed with a 95% confidence interval. $*p < 0.05$ compared to wild-type.

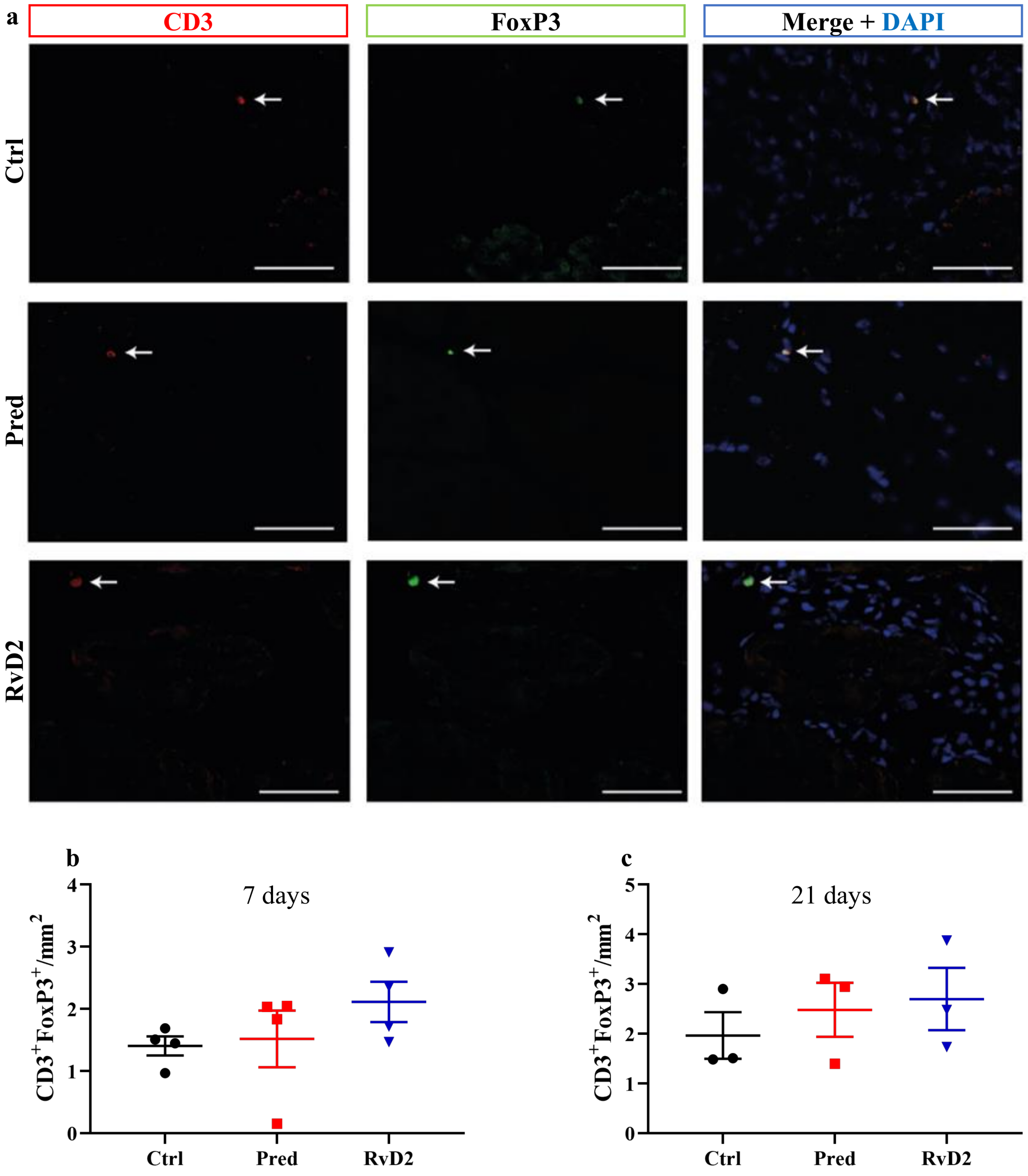

Supplemental Figure 7. Resolvin-D2 does not affect the accumulation of Tregs. a Representative images of *tibialis anterior* (TA) of *mdx* mice treated with daily i.p. injection of prednisone (pred), RvD2, or vehicle. Scale bars = 50 µm. Co-immunofluorescence of CD3 (lymphocyte marker, red), FoxP3 (Treg marker, green), and DAPI (blue). **b,c** Quantification of Treg density ($CD3^{+}FoxP3^{+}$) after (**b**) 7 days, and (**c**) 21 days of treatment. Data are presented as mean ± SEM; n = 3 (panel **c**) or n = 4 (panel **b**) mice per group. Data were analyzed using one-way ANOVA uncorrected Fisher's LSD test. All data were analyzed with a 95% confidence interval.

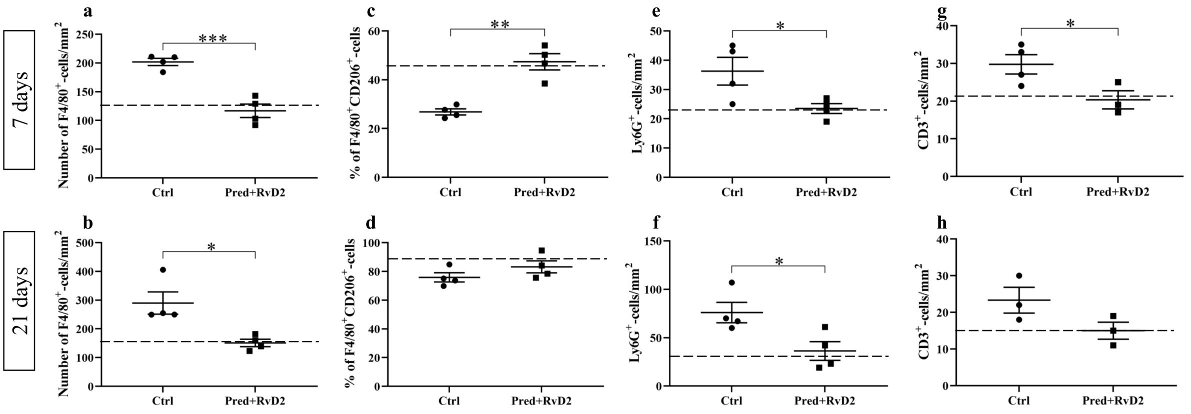

Supplemental Figure 8. Combination of Resolvin-D2 and prednisone does not induce a cumulative anti-inflammatory effect. a-h Daily *ip* injection of both Resolvin-D2 **(**RvD2, 5 ug/kg/day) and prednisone (pred, 2 mg/kg/day) compared to vehicle after 7 days (**a**,**c**,**e**,**g**) and 21 days of treatment (**b**,**d**,**f**,**h**). Dashed lines indicate the value of RvD2 treatment alone (Fig. 4). **a,b** Quantification of total macrophages (F4/80, pan-macrophage marker) and **c,d** percentage of anti-inflammatory macrophages ($F4/80^+CD206^+$ / total $F4/80^+$) in the *tibialis anterior* (TA) muscle of *mdx* mice after 7 days (total macrophages, $p = 0.0007$; anti-inflammatory macrophages, $p = 0.0012$) and 21 days (total macrophages, $p = 0.0143$). **e,f** Density of neutrophils ($Ly6G^+$ cells) in TA muscles following 7 days ($p = 0.0439$) and 21 days ($p=0.0320$). **g,h** Density of T cells ($CD3^+$) in TA muscles following 7 days ($p = 0.0489$) and 21 days. Data are presented as mean ± SEM, n = 4 (except n = 3 for panel **h**, and Pred+RvD2 group in panel **g**) mice per group/time-point. Experiments were performed in technical duplicates and analyzed with the two-tailed unpaired Student's t-test. All data were analyzed with a 95% confidence interval. *$p < 0.05$, **$p < 0.01$, ***$p < 0.001$.

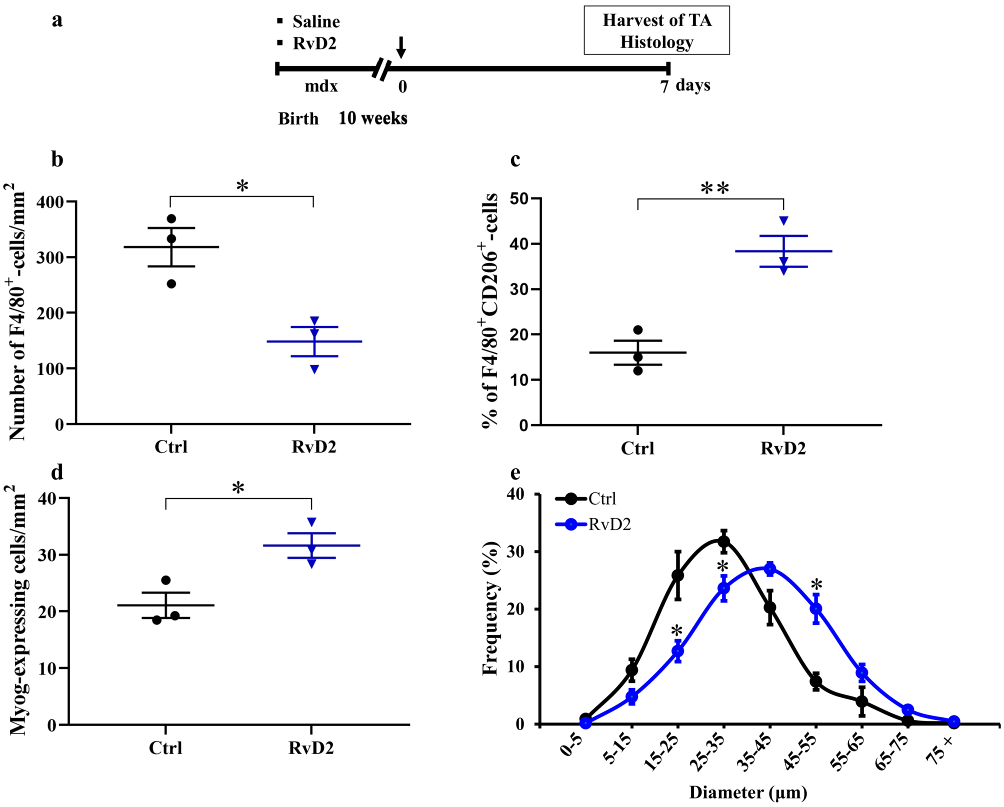

Supplemental Figure 9. Intramuscular administration of Resolvin-D2 dampens inflammation and enhances muscle regeneration in *mdx* mice. a Schematic of the experimental procedure characterized by a single intramuscular injection of Resolvin-D2 (RvD2, 5 μg/kg) or saline (Ctrl) within the *Tibialis anterior* (TA) of *mdx* mice, followed by histological measurements 7 days later. **b-e** Quantification of total macrophage density (F4/80, pan-macrophage marker; p = 0.0173)**(b),** percentage of anti-inflammatory macrophages ($F4/80^+CD206^+$ / total $F4/80^+$; p = 0.0065) (**c**), density of differentiated myoblasts ($Myog^+$; p = 0.0359) (**d**), and muscle fiber diameter of treated mice (RvD2 vs Ctrl: 15-25 μm, p = 0.0443; 25-35 μm, p = 0.0493; 45-55 μm, p = 0.0116) (**e**). Data are presented as mean ± SEM, n = 3 mice per group. Experiments were performed in technical duplicates and analyzed with the two-tailed unpaired Student's t-test. All data were analyzed with a 95% confidence interval. *p < 0.05, **p < 0.01.

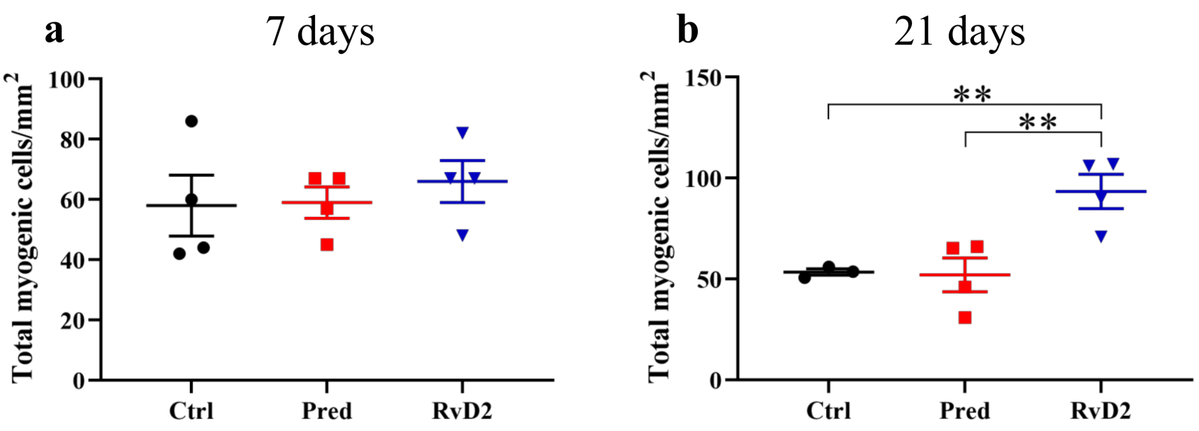

Supplemental Figure 10. Resolvin-D2 increases total myogenic cell number. a,b Total number of myogenic cells ($Pax7^+$ and $Myog^+$ cells) was counted on *tibialis anterior* (TA) muscle sections of *mdx* mice treated with daily injection of prednisone (Pred, 2 mg/kg/day), Resolvin-D2 (RvD2, 5 ug/kg/day), or vehicle (Ctrl) for (**a**) 7 days, or (**b**) 21 days (21d: Ctrl vs. Pred, $p = 0.9026$; Ctrl vs. RvD2, $p = 0.0074$; Pred vs. RvD2, $p = 0.0040$). Data are presented as mean ± SEM, n = 4 (except n = 3 for Ctrl group in panel **b**) mice per group/time-point. Experiments were performed in technical duplicates and analyzed using one-way ANOVA uncorrected Fisher's LSD test. All data were analyzed with a 95% confidence interval. **$p < 0.01$.

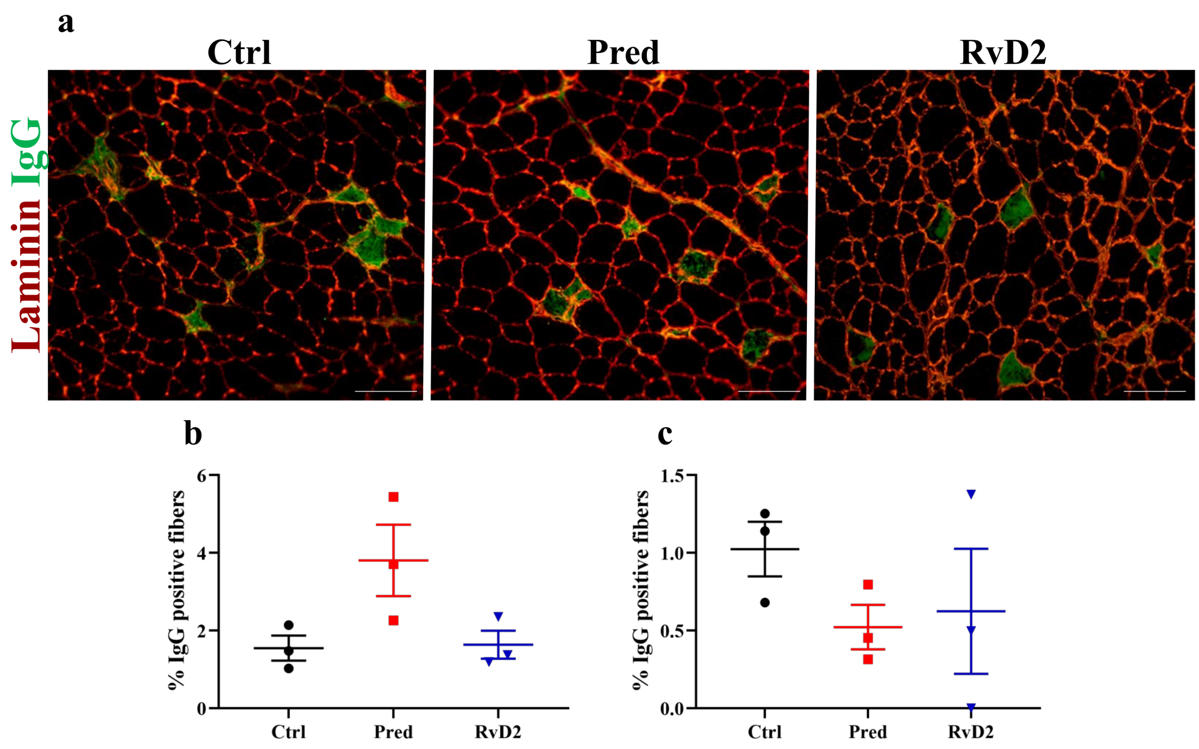

Supplemental Figure 11. Resolvin-D2 has no impact on muscle fiber necrosis in *mdx* mice. **a** Representative images of immunostaining of laminin (red) and IgG (green) on tibialis anterior (TA) of *mdx* mice treated with daily injection with either Resolvin-D2 (RvD2, 5 ug/kg/day), prednisone (pred, 2 mg/kg/day) or vehicle (Ctrl). Scale bars = 100 µm. Percentage of IgG positive necrotic fibers in TA muscle sections of *mdx* mice following (**b**) 7 days, and (**c**) 21 days of treatment. Data are presented as mean ± SEM; n = 3 mice per group/time-point. Data were analyzed using one-way ANOVA uncorrected Fisher's LSD test. All data were analyzed with a 95% confidence interval.

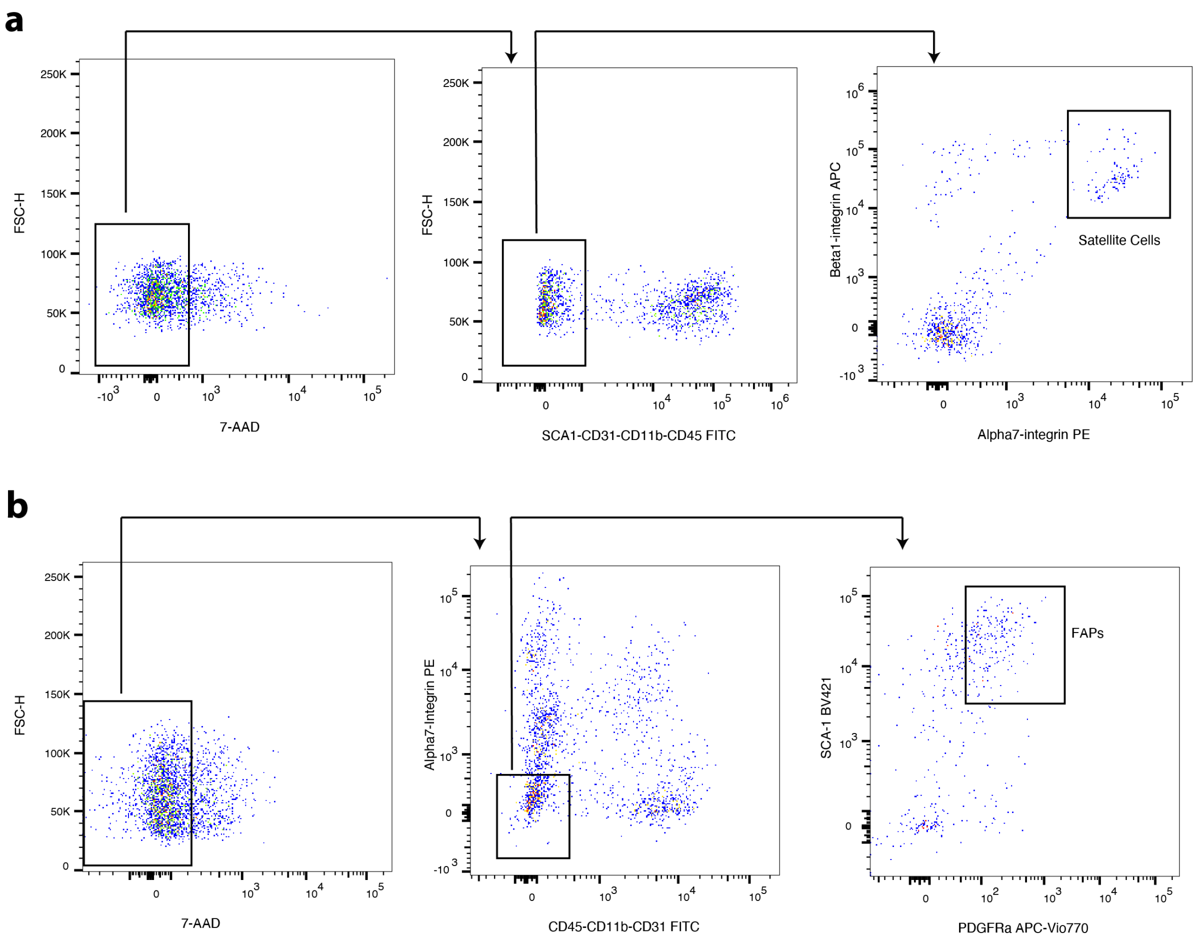

Supplemental figure 12: FACS gating strategies. a Strategies for cell sorting satellite cells from mouse hindlimb skeletal muscles (cells used in Fig 1-3 and Suppl Fig. 2-6). Cells were sorted based on forward scatter (FSC-H), cell viability (7-AAD), negative selection with FITC-conjugated antibodies for anti-Sca-1, anti-CD45, anti-CD31, anti-CD11b, and positive selection for APC-conjugated anti-Itgb1 and PE-conjugated anti-Itga7. **b** Strategies for cell sorting fibroadipogenic progenitors (FAPs) from mouse hindlimb skeletal muscles (cells used in Suppl Fig. 5). Cells were sorted using a gating strategy based on forward scatter (FSC-H) and cell viability (7-AAD), followed by negative selection with FITC-conjugated antibodies for anti-CD45, anti-CD31, anti-CD11b, and PE-conjugated anti-Itga7, and positive selection for APC-Vio 770 anti-CD140a (pdgfra) and BV421 anti-Sca-1.

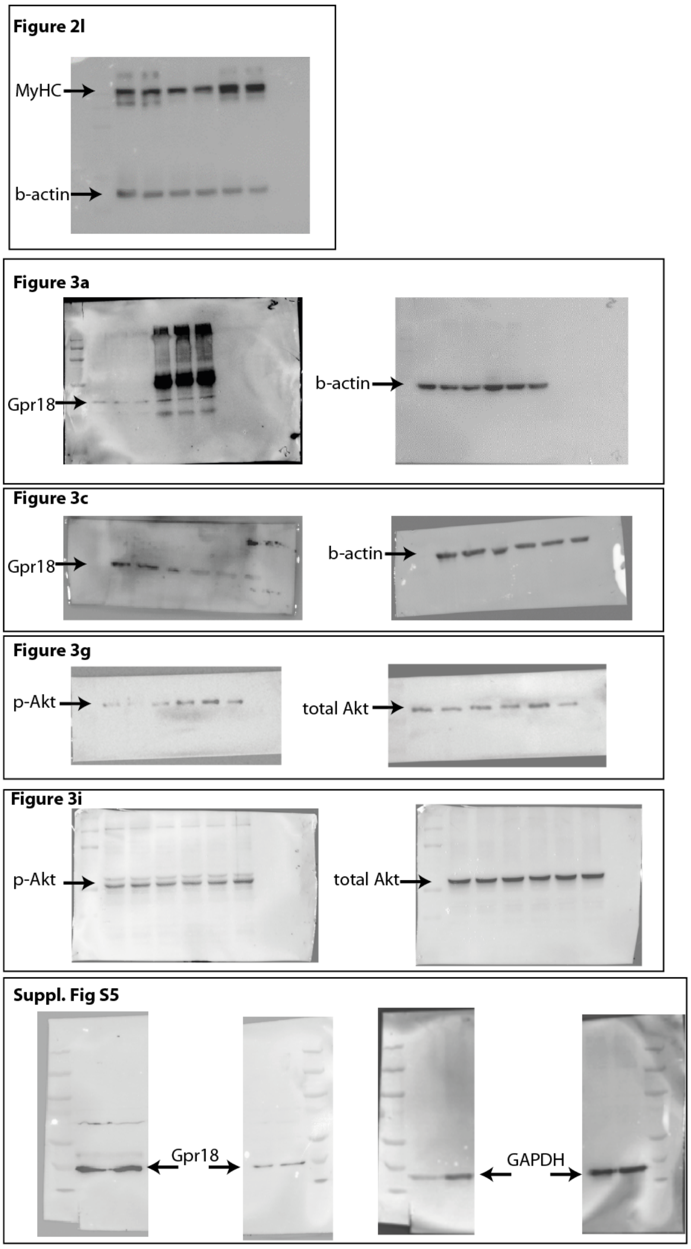

Supplemental Figure 13. Uncropped gels of Western blot shown in main and supplemental figures.

**Genes**	**Sequences**
Tnf	forward: 5'-AGCCGATGGGTTGTACCTTG-3'<br>reverse: 5'-CTCCAAAGTAGACCTGCCCG-3'
Il-4	forward: 5'-CAGCAACGAAGAACACCACAG-3'<br>reverse: 5'-AAGCCCGAAAGAGTCTCTGC-3'
Cxcl1	forward: 5'-TGGCTGGGATTCACCTCAAG-3'<br>reverse: 5'-AGTGTGGCTATGACTTCGGTT-3'
Tgfb1	forward: 5'-ACCGCAACAACGCCATCTAT-3'<br>reverse: 5'-TGCCGTACAACTCCAGTGAC-3'
Anxa1	forward: 5'-GGTGACCGTTGTCAGGACTT-3'<br>reverse: 5'-CTGGTGGCACACTTCACGAT-3'
Cd163	forward: 5'-GAGACACACGGAGCCATCAA-3'<br>reverse: 5'- TGGACAAACCTTTTACAACCAGG-3'
Pparg	forward: 5'-GGTGAACCACTGATATTCAGGACA-3'<br>reverse: 5'-GTTCTACTTTGATCGCACTTTGGT-3'
Chil3	forward: 5'-GAAGCTCTCCAGAAGCAATCCTG-3'<br>reverse: 5'- TTCAGAAGAATTGCCAGACCTGT-3'
Gpr18	forward: 5'-CTGAAGCCCAAGGTCAAGGA-3'<br>reverse: 5'-TTGTAGCATCAGGACGGCAA-3'
Ptgs2	forward: 5'-CATCCCCTTCCTGCGAAGTT-3'<br>reverse: 5'-CATGGGAGTTGGGCAGTCAT-3'
Ptges	forward: 5'-CCTGGAAGGGAACTTTGGCT-3'<br>reverse: 5'-TCAGGACTCTGGAGGGACAC-3'
Cd80	forward: 5'-TGCTCTCAGAACCAAGCCAC-3'<br>reverse: 5'-ATGCTGCAGCTTACTTCCCC-3'
Gapdh	forward: 5’-CCCAGAAGACTGTGGATGG-3’<br>reverse: 5’- ACACATTGGGGGTAGGAACA -3'

Supplemental table 1. List of primers used for qPCR experiments.

Compounds	Standard (m/z)	Internal standards (m/z)
**Leukotriene**		
LTB_4	335.3 → 195.1	339.3 → 197.1
**Resolvins**		
RvD1	375.2 → 141.0	380.3 → 141.0
RvD2	375.3 → 175.1	380.4 → 175.0
RvD3	375.3 → 147.2	
17(*R,S*)-RVD4	375.2 → 101.0	
RvD5	359.3 → 199.0	
RvE1	349.2 → 195.0	
**Prostaglandins**		
TxB_2	369.3 → 195.0	373.3 → 199.1
$PGF_{2\alpha}$	353.3 → 193.0	357.3 → 197.2
PGE_2	351.2 → 189.0	360.2→ 189.0

Supplemental table 2. Selected reaction monitoring transitions for tandem mass spectrometry. TXB_2 (thromboxane-B_2), LTB_4 (Leukotriene-B_4), PGE_2 (Prostaglandin-E_2), $PGF_{2\alpha}$ (Prostaglandin-$F_{2\alpha}$), Resolvin-D1 (RvD1), RvD2, RvD3, 17(*R,S*)-RvD4, RvD5, Resolvin-E1 (RvE1).
