# Supplementary material for: Resolvin-D2 targets myogenic cells and improves muscle regeneration in Duchenne muscular dystrophy
Source: Nat Commun. 2021 Oct 29;12:6264. doi: 10.1038/s41467-021-26516-0 (PMC8556273; doi:10.1038/s41467-021-26516-0)
Supplement: Supplementary file 2 — Reporting Summary [file 41467_2021_26516_MOESM2_ESM.pdf]

nature research

Corresponding author(s): Nicolas A. Dumont

Last updated by author(s): Jul 14, 2021

Reporting Summary

Nature Research wishes to improve the reproducibility of the work that we publish. This form provides structure for consistency and transparency in reporting. For further information on Nature Research policies, see our Editorial Policies and the Editorial Policy Checklist.

Statistics

For all statistical analyses, confirm that the following items are present in the figure legend, table legend, main text, or Methods section.

n/a	Confirmed	
☐	☒	The exact sample size (n) for each experimental group/condition, given as a discrete number and unit of measurement
☐	☒	A statement on whether measurements were taken from distinct samples or whether the same sample was measured repeatedly
☐	☒	The statistical test(s) used AND whether they are one- or two-sided<br>*Only common tests should be described solely by name; describe more complex techniques in the Methods section.*
☐	☒	A description of all covariates tested
☐	☒	A description of any assumptions or corrections, such as tests of normality and adjustment for multiple comparisons
☐	☒	A full description of the statistical parameters including central tendency (e.g. means) or other basic estimates (e.g. regression coefficient) AND variation (e.g. standard deviation) or associated estimates of uncertainty (e.g. confidence intervals)
☐	☒	For null hypothesis testing, the test statistic (e.g. F, t, r) with confidence intervals, effect sizes, degrees of freedom and P value noted<br>*Give P values as exact values whenever suitable.*
☒	☐	For Bayesian analysis, information on the choice of priors and Markov chain Monte Carlo settings
☒	☐	For hierarchical and complex designs, identification of the appropriate level for tests and full reporting of outcomes
☒	☐	Estimates of effect sizes (e.g. Cohen's d, Pearson's r), indicating how they were calculated

Our web collection on statistics for biologists contains articles on many of the points above.

Software and code

Policy information about availability of computer code

Data collection	Analyst software (version 1.7)
Data analysis	FlowJo software (v10.6.2), ImageJ Software (version 1.53), GeneSys image software (version), SCIEX OS software (version 1.7MQ)

For manuscripts utilizing custom algorithms or software that are central to the research but not yet described in published literature, software must be made available to editors and reviewers. We strongly encourage code deposition in a community repository (e.g. GitHub). See the Nature Research guidelines for submitting code & software for further information.

Data

Policy information about availability of data

All manuscripts must include a data availability statement. This statement should provide the following information, where applicable:

- Accession codes, unique identifiers, or web links for publicly available datasets
- A list of figures that have associated raw data
- A description of any restrictions on data availability

A data availability statement is included in the manuscript: "The raw data generated for all figures (Fig. 1-8 and Suppl. Fig. 1-11) of this study are provided in a Supplementary Excel file."

Field-specific reporting

Life sciences study design

All studies must disclose on these points even when the disclosure is negative.

Sample size	Sample size determination was based on the expected effect size and variability that was previously observed by the investigators for similar readouts using mdx mice (PMID: 26569381, PMID: 30713094).
Data exclusions	No data was excluded.
Replication	All experiments were repeated independently at least twice in the laboratory with similar results
Randomization	Mice from the same litter were randomly assigned to different experimental groups.
Blinding	Experimenters were blinded to the identity of the sample during data collection and analysis.

Reporting for specific materials, systems and methods

We require information from authors about some types of materials, experimental systems and methods used in many studies. Here, indicate whether each material, system or method listed is relevant to your study. If you are not sure if a list item applies to your research, read the appropriate section before selecting a response.

Materials & experimental systems

n/a	Involved in the study
☐	☒ Antibodies
☐	☒ Eukaryotic cell lines
☒	☐ Palaeontology and archaeology
☐	☒ Animals and other organisms
☒	☐ Human research participants
☒	☐ Clinical data
☒	☐ Dual use research of concern

Methods

n/a	Involved in the study
☒	☐ ChIP-seq
☐	☒ Flow cytometry
☒	☐ MRI-based neuroimaging

Antibodies

Antibodies used

Mouse anti-Pax7 (clone PAX7, 1:20; DSHB) and rabbit anti-myogenin (clone EPR4789; 1:500; Abcam), mouse anti-Myh3 (clone F1.652; 0.3 ug/ml; DSHB) and rabbit anti-Laminin (cat# ab11575; 1:1,000; Abcam), rat anti-F4/80 monoclonal antibody (clone A3-1; 1:1,000; Bio-Rad) and rabbit CD206 monoclonal antibody (cat# ab64693; 1:2,000; Abcam), rat anti-Ly-6G monoclonal antibody (clone 1A8; 1:250; Invitrogen), rabbit CD3 monoclonal antibody (clone SP7; 1:1,000; Abcam), mouse anti-myosin heavy chain (clone MF20; 1:20; DSHB), rat anti-mouse CD25-PE conjugated (Clone PC61; 1:40; BD Bioscience), F4/80-FITC conjugated (clone REA126; 1:50; Miltenyi Biotec), rat anti-mouse CD163-PE conjugated (clone TNKUPJ; 1:50; Thermo Fisher Scientific), rat anti-mouse arginase-APC conjugated (clone A1exF5; 1:50; Thermo Fisher Scientific), rat Ki67 monoclonal antibody (clone SolA15, 5μg/ml, Thermo Fisher Scientific), or rabbit polyclonal anti-Gpr18 (1:1,000; Sigma). mouse anti-myosin heavy chain (clone MF20; 1:20, DSHB), rabbit anti-Gpr18 (cat# SAB4501253; 1:1,000; Sigma), rabbit anti-(pan)-Akt (clone C67E7; 1,1000; Cell Signaling technology), rabbit anti-phospho-Akt (Ser473) (cat# 9271; 1:1,000; Cell Signaling technology), rabbit anti-b-actin (cat# 4967; 1:1,000; Cell Signaling Technology), or rabbit anti-GAPDH (cat# 2118; 1:1000; Cell Signaling Technology). FITC-conjugated anti-CD45 (clone 30F11, 1:30, Miltenyi Biotech), FITC-conjugated anti-CD31 (Clone 390, 1:30, Miltenyi Biotech), FITC-conjugated anti-CD11b (M1/70.15.11.5, 1:30, Miltenyi Biotech), APC-conjugated anti-Itgb1 (clone HMβ1-1, 1:15, Miltenyi Biotech), PE-conjugated anti-Itga7 (clone 3C12, 1:100, Miltenyi Biotech), APC-Vio 770 anti-CD140a (Clone REA637, 1:15, Miltenyi Biotech), BV421 anti-Sca-1 (Clone D7, 1:100, Miltenyi Biotech), F4/80-FITC conjugated (clone REA126, 1:50, Miltenyi Biotec), iNOS-PE-conjugated anti-mouse antibody (clone REA982, 1:50, Miltenyi Biotec).

Validation

• Mouse anti-Pax7 (clone PAX7, 1:20; DSHB): This Ab has been deposited to the Developmental Studies Hybridoma Bank by Kawakami, A. It has been validated on mouse tongue muscle by immunofluorescence. Multiple publications describe this Ab. Selected reference: Seale, P., Sabourin, L.A., Girgis-Gabardo, A., Mansouri, A., Gruss, P., and Rudnicki, M.A. (2000). Pax7 is required for the specification of myogenic satellite cells. Cell 102:777-786.
• rabbit anti-myogenin (clone EPR4789; 1:500; Abcam). This Ab has been validated by immunofluorescence and western blot by the manufacturer on muscle extract, mouse myoblast, and Human iPSC-Derived Skeletal Myocytes. Multiple publications describe this Ab. Selected ref: Akiyama T, Sato S, Chikazawa-Nohtomi N, Soma A, Kimura H, Wakabayashi S, Ko SBH, Ko MSH. Efficient differentiation of human pluripotent stem cells into skeletal muscle cells by combining RNA-based MYOD1-expression and POU5F1-silencing. Sci Rep. 2018 Jan 19;8(1):1189.
• anti-Myh3 (clone F1.652; 0.C3 ug/ml; DSHB)): This Ab has been deposited to the Developmental Studies Hybridoma Bank by Blau HM Lab from Stanford University. It has been validated by immunofluorescence on regenerating mouse skeletal muscles. Multiple publications describe this Ab. Selected reference: Lu A, Guo P, Pan H, Tseng C, Sinha KM, Yang F, Scibetta A, Cui Y, Huard M, Zhong L, Ravuri S, Huard J. Enhancement of myogenic potential of muscle progenitor cells and muscle healing during pregnancy. FASEB J. 2021 Mar;35(3):e21378.
• rabbit anti-Laminin (cat# ab11575; 1:1,000; Abcam). This Ab has been validated by the manufacturer by immunofluorescence on human tongue section and Western blot on murine sarcoma basement membrane. Multiple publications describe this Ab. Selected

reference : Scott RW, Arostegui M, Schweitzer R, Rossi FMV, Underhill TM. Hic1 Defines Quiescent Mesenchymal Progenitor Subpopulations with Distinct Functions and Fates in Skeletal Muscle Regeneration. Cell Stem Cell. 2019 Dec 5;25(6):797-813.
• rat anti-F4/80 monoclonal antibody (clone A3-1; 1:1,000; Bio-Rad). This Ab has been validated by immunofluorescence by multiple publications. Selected reference : Muto A, Yi T, Harrison KD, Dávalos A, Fancher TT, Ziegler KR, Feigel A, Kondo Y, Nishibe T, Sessa WC, Dardik A. Eph-B4 prevents venous adaptive remodeling in the adult arterial environment. J Exp Med. 2011 Mar 14;208(3):561-75.
• rabbit CD206 monoclonal antibody (cat# ab64693; 1:2,000; Abcam): This Ab has been validated by the manufacturer by immunofluorescence and western blot on mouse lung tissue. Multiple publications describe this Ab. Selected reference: Yin J, Kim SS, Choi E, Oh YT, Lin W, Kim TH, Sa JK, Hong JH, Park SH, Kwon HJ, Jin X, You Y, Kim JH, Kim H, Son J, Lee J, Nam DH, Choi KS, Shi B, Gwak HS, Yoo H, Iavarone A, Kim JH, Park JB. ARS2/MAGL signaling in glioblastoma stem cells promotes self-renewal and M2-like polarization of tumor-associated macrophages. Nat Commun. 2020 Jun 12;11(1):2978.
• rat anti-Ly-6G monoclonal antibody (clone 1A8; 1:250; Invitrogen). This Ab has been validated by immunofluorescence by multiple publications. Selected reference : Hiroki CH, Toller-Kawahisa JE, Fumagalli MJ, Colon DF, Figueiredo LTM, Fonseca BALD, Franca RFO, Cunha FQ. Neutrophil Extracellular Traps Effectively Control Acute Chikungunya Virus Infection. Front Immunol. 2020 Jan 31;10:3108.
• rabbit CD3 monoclonal antibody (clone SP7; 1:1,000; Abcam). This Ab has been validated by the manufacturer by immunofluorescence on rat infarcted heart tissue and by western blot on mouse thymus lysate. Multiple publications describe this Ab. Selected reference : Nagase T, Ueno K, Mizoguchi T, Samura M, Harada T, Suehiro K, Shirasawa B, Morikage N, Hamano K. Allogeneic fibroblast sheets accelerate cutaneous wound healing equivalent to autologous fibroblast sheets in mice. Am J Transl Res. 2020 Jun 15;12(6):2652-2663. PMID: 32655797; PMCID: PMC7344096.
• mouse anti-myosin heavy chain (clone MF20; 1:20; DSHB): The manufacturer has validated this Ab by immunofluorescence of C2C12 mouse cells and immunohistochemistry of human skeletal muscle. Multiple publications describe this Ab for immunostaining and western blot. Selected reference: Fujimaki S, Seko D, Kitajima Y, Yoshioka K, Tsuchiya Y, Masuda S, Ono Y. Notch1 and Notch2 Coordinately Regulate Stem Cell Function in the Quiescent and Activated States of Muscle Satellite Cells. Stem Cells. 2018 Feb;36(2):278-285.
• rat anti-mouse CD25-PE conjugated (Clone PC61; 1:40; BD Bioscience) This Ab has been validated by the manufacturer by Flow cytometry on mouse bone marrow. Multiple publications describe this Ab. Selected ref : Read S, Malmström V, Powrie F. Cytotoxic T lymphocyte-associated antigen 4 plays an essential role in the function of CD25(+)CD4(+) regulatory cells that control intestinal inflammation. J Exp Med. 2000 Jul 17;192(2):295-302.
• rat anti-mouse CD163-PE conjugated (clone TNKUPJ; 1:50; Thermo Fisher Scientific). This Ab has been validated by the manufacturer by immunofluorescence on mouse spleen and liver. Multiple publications describe this Ab. Selected reference : Gyori D, Lim EL, Grant FM, Spensberger D, Roychoudhuri R, Shuttleworth SJ, Okkenhaug K, Stephens LR, Hawkins PT. Compensation between CSF1R+ macrophages and Foxp3+ Treg cells drives resistance to tumor immunotherapy. JCI Insight. 2018 Jun 7;3(11):e120631.
• rat anti-mouse arginase-APC conjugated (clone A1exF5; 1:50; Thermo Fisher Scientific). This Ab has been validated by flow cytometry by the manufacturer on mouse bone marrow derived macrophages polarized to M1 (negative control) or M2 (positive control). Multiple publications describe this Ab. Selected reference : Wiktorowicz JE, Chowdhury IH, Stafford S, Choudhuri S, Dey N, Garg NJ. Integrated Functional Analysis of the Nuclear Proteome of Classically and Alternatively Activated Macrophages. Mediators Inflamm. 2019 Apr 30;2019:3481430.
• rat Ki67 monoclonal antibody (clone SolA15, 5µg/ml, Thermo Fisher Scientific). This Ab has been validated by immunostaining by the manufacturer on C2C12 myoblasts. Multiple publications describe this Ab. Selected reference : Akram KM, Yates LL, Mongey R, Rothery S, Gaboriau DCA, Sanderson J, Hind M, Griffiths M, Dean CH. Live imaging of alveologenesis in precision-cut lung slices reveals dynamic epithelial cell behaviour. Nat Commun. 2019 Mar 12;10(1):1178.
• rabbit anti-Gpr18 (cat# SAB4501253; 1:1,000; Sigma): This antibody has been validated by the manufacturer for immunofluorescence and western blot on HuvEc and Jurkat cells. Negative controls were used with both anti-Gpr18 antibody and the synthesized immunogen peptide. We further validated this antibody using siRNA knockdown for Gpr18 (Fig 3C). Multiple publications describe this Ab. Selected reference: Flegel C, Vogel F, Hofreuter A, Wojcik S, Schoeder C, Kieć-Kononowicz K, Brockmeyer NH, Müller CE, Becker C, Altmüller J, Hatt H, Gisselmann G. Characterization of non-olfactory GPCRs in human sperm with a focus on GPR18. Sci Rep. 2016 Aug 30;6:32255.
• rabbit anti-(pan)-Akt (clone C67E7; 1,1000; Cell Signaling technology). This antibody has been validated by the manufacturer for immunostaining and western blot on various cell lines (e.g. HeLa, NIH/3T3). Multiple publications describe this Ab. Selected reference: Guarnaccia AD, Rose KL, Wang J, Zhao B, Popay TM, Wang CE, Guerrazzi K, Hill S, Woodley CM, Hansen TJ, Lorey SL, Shaw JG, Payne WG, Weissmiller AM, Olejniczak ET, Fesik SW, Liu Q, Tansey WP. Impact of WIN site inhibitor on the WDR5 interactome. Cell Rep. 2021 Jan 19;34(3):108636.
• rabbit anti-phospho-Akt (Ser473) (cat# 9271; 1:1,000; Cell Signaling technology). This antibody has been validated by western blot by the manufacturer on extracts from NIH/3T3 cells, untreated or treated with PDGF and/or Akt/mTOR inhibitors Multiple publications describe this Ab. Selected reference: van Drongelen V, Scavuzzi BM, Nogueira SV, Miller FW, Sawalha AH, Holoshitz J. HLA-DRB1 allelic epitopes that associate with autoimmune disease risk or protection activate reciprocal macrophage polarization. Sci Rep. 2021 Jan 28;11(1):2599.
• rabbit anti-b-actin (cat# 4967; 1:1,000; Cell Signaling Technology). This antibody has been validated by western blot by the manufacturer on extracts from different cell lines, including C2C12 myoblasts. Multiple publications describe this Ab. Selected reference: Gordon DE, Hiatt J, Bouhaddou M, Rezelj VV, Ulferts S, Braberg H, et al. Comparative host-coronavirus protein interaction networks reveal pan-viral disease mechanisms. Science. 2020 Dec 4;370(6521):eabe9403.
• rabbit anti-GAPDH (cat# 2118; 1:1000; Cell Signaling Technology). This antibody has been validated by western blot by the manufacturer on extracts from different cell lines (e.g. NIH/3T3, HUVEC, Hela, C6). Multiple publications describe this Ab. Selected reference: Osei-Owusu J, Yang J, Leung KH, Ruan Z, Lü W, Krishnan Y, Qiu Z. Proton-activated chloride channel PAC regulates endosomal acidification and transferrin receptor-mediated endocytosis. Cell Rep. 2021 Jan 26;34(4):108683.
• FITC-conjugated anti-CD45 (clone 30F11, Miltenyi Biotech). This antibody has been validated by flow cytometry by the manufacturer on mouse splenocytes Multiple publications describe this Ab. Selected reference: Podd BS, Thoits J, Whitley N, Cheng HY, Kudla KL, Taniguchi H, Halkias J, Goth K, Camerini V. T cells in cryptopatch aggregates share TCR gamma variable region junctional sequences with gamma delta T cells in the small intestinal epithelium of mice. J Immunol. 2006 Jun 1;176(11):6532-42.
• FITC-conjugated anti-CD31 (Clone 390, Miltenyi Biotech). This antibody has been validated by flow cytometry by the manufacturer on mouse splenocytes Multiple publications describe this Ab. Selected reference: Wang X, Li F, Xie L, Crane J, Zhen G, Mishina Y, Deng R, Gao B, Chen H, Liu S, Yang P, Gao M, Tu M, Wang Y, Wan M, Fan C, Cao X. Inhibition of overactive TGF-β attenuates progression of heterotopic ossification in mice. Nat Commun. 2018 Feb 7;9(1):551.
• FITC-conjugated anti-CD11b (M1/70.15.11.5, Miltenyi Biotech). This antibody has been validated by flow cytometry by the

manufacturer on mouse splenocytes Multiple publications describe this Ab. Selected reference: Winsauer C, Prepens S, Schlienz D, Nedospasov S, Kruglov AA. Novel mouse model to study T cell-dependent IgA induction in vivo. J Immunol Methods. 2015 Jun;421:54-60.
• APC-conjugated anti-Itgb1 (clone HMβ1-1, Miltenyi Biotech). This antibody has been validated by flow cytometry by the manufacturer on mouse splenocytes Multiple publications describe this Ab. Selected reference: Yu B, Huo L, Liu Y, Deng P, Szymanski J, Li J, Luo X, Hong C, Lin J, Wang CY. PGC-1α Controls Skeletal Stem Cell Fate and Bone-Fat Balance in Osteoporosis and Skeletal Aging by Inducing TAZ. Cell Stem Cell. 2018 Oct 4;23(4):615-623.
• PE-conjugated anti-Itga7 (clone 3C12, Miltenyi Biotech). This antibody has been validated by flow cytometry by the manufacturer on C2C12 myoblasts. Multiple publications describe this Ab. Selected reference: Engl T, Rutz J, Maxeiner S, Fanguen S, Juengel E, Koschade S, Roos F, Khoder W, Tsaur I, Blaheta RA. Acquired resistance to temsirolimus is associated with integrin α7 driven chemotactic activity of renal cell carcinoma in vitro. Oncotarget. 2018 Apr 10;9(27):18747-18759. doi: 10.18632/oncotarget.24650. PMID: 29721158; PMCID: PMC5922352.
• APC-Vio-conjugated 770 anti-CD140a (Clone REA637, Miltenyi Biotech). This antibody has been validated by flow cytometry by the manufacturer on 3T3-A31 cells. Double staining with another clone of recognizing the same antigen revealed complete overlap. Multiple publications describe this Ab. Selected reference: McGowan SE, McCoy DM. Neuropilin-1 and platelet-derived growth factor receptors cooperatively regulate intermediate filaments and mesenchymal cell migration during alveolar septation. Am J Physiol Lung Cell Mol Physiol. 2018 Jul 1;315(1):L102-L115.
• BV421-conjugated anti-Sca-1 (Clone D7, BioLegend) This antibody has been validated by flow cytometry by the manufacturer on mouse splenocytes. Multiple publications describe this Ab. Selected reference: LaFleur MW, Nguyen TH, Coxe MA, Yates KB, Trombley JD, Weiss SA, Brown FD, Gillis JE, Coxe DJ, Doench JG, Haining WN, Sharpe AH. A CRISPR-Cas9 delivery system for in vivo screening of genes in the immune system. Nat Commun. 2019 Apr 10;10(1):1668.
• F4/80-FITC conjugated (clone REA126, 1:50, Miltenyi Biotec) This antibody has been validated by flow cytometry by the manufacturer on mouse splenocytes Multiple publications describe this Ab. Selected reference: Gu CJ, Borjabad A, Hadas E, Kelschenbach J, Kim BH, Chao W, Arancio O, Suh J, Polsky B, McMillan J, Edagwa B, Gendelman HE, Potash MJ, Volsky DJ. EcoHIV infection of mice establishes latent viral reservoirs in T cells and active viral reservoirs in macrophages that are sufficient for induction of neurocognitive impairment. PLoS Pathog. 2018 Jun 7;14(6):e1007061.
• iNOS-PE-conjugated anti-mouse antibody (clone REA982, 1:50, Miltenyi Biotec). This antibody has been validated by flow cytometry by the manufacturer on peritoneal exudate cells activated with LPS. Multiple publications describe this Ab. Selected reference: Zaidi NE, Shazali NAH, Chor ALT, Osman MA, Ibrahim K, Jaoi-Edward M, Afizan Nik Abd Rahman NM. Time-Lapse 2D Imaging of Phagocytic Activity in M1 Macrophage-4T1 Mouse Mammary Carcinoma Cells in Co-cultures. J Vis Exp. 2019 Dec 14;(154). doi: 10.3791/60281. PMID: 31885381.

Eukaryotic cell lines

Policy information about cell lines

Cell line source(s)	primary cells
Authentication	N/A
Mycoplasma contamination	All cell line tested negative for mycoplasma contamination.
Commonly misidentified lines (See ICLAC register)	N/A

Animals and other organisms

Policy information about studies involving animals; ARRIVE guidelines recommended for reporting animal research

Laboratory animals	Experiments were performed on ten-week-old mdx mice and six-week-old mdx-utrn dKO mice. Only male mice were used. Mice were housed on a 12:12 h light:dark cycle in pathogen-free cages at 21 C and 40% humidity
Wild animals	no wild animals were used in the study.
Field-collected samples	no field collected samples were used in the study.
Ethics oversight	All animal experiments were approved by the CHU Sainte-Justine Research Ethics Committee and performed in compliance with the Comité Institutionnel des Bonnes Pratiques Animales en Recherche (CIBPAR; approval number 2020-2668) in accordance with the Canadian Council on Animal Care guidelines

Note that full information on the approval of the study protocol must also be provided in the manuscript.

Flow Cytometry

Plots

Confirm that:

- [x] The axis labels state the marker and fluorochrome used (e.g. CD4-FITC).
- [x] The axis scales are clearly visible. Include numbers along axes only for bottom left plot of group (a 'group' is an analysis of identical markers).
- [x] All plots are contour plots with outliers or pseudocolor plots.
- [x] A numerical value for number of cells or percentage (with statistics) is provided.

Methodology

Sample preparation	For satellite cells and fibroadipogenic progenitors, Muscles from both hindlimbs of mdx mice were collected and dissociated in collagenase/dispase (Sigma) using the gentle MACS dissociator (Miltenyi Biotech), filtered through a 30 um cell strainer, and stained with antibodies for 30 min on ice in the dark.<br>For macrophages: Single cell suspensions were washed and incubated for 30 min at 4°C with the eBioscience Fixable Live-Dead Viability Dye Fluor 506 (Ottawa, ON, Canada). Cell suspensions were washed, incubated with 10% FcR block for 15 min, and labeled for 30 min at 4°C with, F4/80-FITC conjugated diluted at 1:50 (clone REA126, Miltenyi Biotec, California, USA). Samples were washed, fixed with 2% PFA (in PBS 1X) for 5 min and permeabilized with 0.2% Triton X-100 for 10 min. Thereafter, samples were incubated for 30 min at 4°C with the intracellular iNOS-PE-conjugated anti-mouse antibody (clone REA982, 1:50 in permeabilization buffer, Miltenyi Biotec)
Instrument	LSR Fortessa flow cytometer
Software	FlowJo v10.6.2 software
Cell population abundance	For satellite cells and fibroadipogenic progenitors the cell population abundance is roughly 1% of the total cell population, which is similar to what has been published before (PMID: 30771188). For macrophages, >99% were positive for the macrophage marker F4/80
Gating strategy	For satellite cell sorting: MuSC were isolated by FACS using a gating strategy based on forward scatter and side scatter profiles, cell viability (7-AAD; 1:40; Biolegend), negative selection with FITC-conjugated antibodies for anti-Sca-1 (Clone D7; 1:30; Miltenyi Biotech), anti-CD45 (clone 30F11; 1:30; Miltenyi Biotech), anti-CD31 (Clone 390; 1:30; Miltenyi Biotech), anti-CD11b (M1/70.15.11.5; 1:30; Miltenyi Biotech), and positive selection for APC-conjugated anti-Itgb1 (clone HMβ1-1; 1:15; Miltenyi Biotech) and PE-conjugated anti-Itga7 (clone 3C12, 1:100; Miltenyi Biotech).<br>For fibroadipogenic progenitors sorting: gating strategy based on forward scatter and side scatter profiles, cell viability (7-AAD; 1:40; Biolegend), negative selection with FITC-conjugated antibodies for anti-CD45 (clone 30F11; 1:30; Miltenyi Biotech), anti-CD31 (Clone 390; 1:30; Miltenyi Biotech), anti-CD11b (M1/70.15.11.5; 1:30; Miltenyi Biotech), PE-conjugated anti-Itga7 (clone 3C12; 1:100; Miltenyi Biotech) and positive selection for APC-Vio 770 anti-CD140a (Clone REA637; 1:15; Miltenyi Biotech) and BV421 anti-Sca-1 (Clone D7; 1:100; Biolegend).<br>For macrophages: F4/80-FITC conjugated (clone REA126, Miltenyi Biotec, California, USA) and iNOS-PE-conjugated anti-mouse antibody (clone REA982, Miltenyi Biotec)

- [x] Tick this box to confirm that a figure exemplifying the gating strategy is provided in the Supplementary Information.
